# Sexual dysfunction and mental health in patients with multiple sclerosis and epilepsy

**DOI:** 10.1186/s12883-020-1625-7

**Published:** 2020-01-31

**Authors:** Marian Petersen, Ellids Kristensen, Laura Giraldi, Annamaria Giraldi

**Affiliations:** 1 The Department of Regional Health Research, University of Southern Denmark, Odense, Denmark; 2 Surgical Department, Zealand University Hospital, Køge, Denmark; 3 Sexological Clinic, Psychiatric Centre Copenhagen & Department of Clinical Medicine, University of Copenhagen, Copenhagen, Denmark; 4 Neuroscience Centre, Rigshospital, Copenhagen, Denmark

**Keywords:** Epilepsy, Multiple sclerosis, Sexuality, Quality of life, Sexual dysfunction

## Abstract

**Background:**

Epilepsy and multiple sclerosis (MS) are two neurological diseases known to greatly influence a patient’s life. The primary aim of this study was to describe the prevalence of sexual dysfunction in patients with epilepsy and MS and investigate whether there is an association between disease, sexual function, and physical and mental health. A secondary aim was to investigate whether there is a difference in sexual function between patients with MS and epilepsy.

**Methods:**

A total of 414 patients were included in this descriptive cross-sectional study. Three patient report questionnaires were used for measurements: the Changes in Sexual Function Questionnaire (CSFQ) cut-off score; the Short Form 36 Health Survey (SF-36) divided into the Physical Component Summary (PCS) and Mental Component Summary (MCS), and the Life Satisfaction—11 (LiSat-11).

**Results:**

Patients with MS constituted 62% (*n* = 258) of the participants and patients with epilepsy 38% (*n* = 156). The prevalence of sexual dysfunction was 68% in women and 77% in men. No differences were found between patients with MS and epilepsy (*p* = 0.184), except for the CSFQ desire domain, as patients with epilepsy more often had a desire problem (*p* = 0.029). On the SF-36, patients with MS scored significantly worse on the PCS (*p* = 0.000). Patients with epilepsy scored significantly worse on the MCS (*p* = 0.002). No significant differences were found on the LiSat-11. Regression analysis with CSFQ as the dependent variable showed an association with the PCS in men and an association with both PCS and MCS in women.

**Conclusions:**

In this study, the cohort of patients with MS and epilepsy had negatively affected sexual function. The only significant difference between patients with MS and epilepsy in sexual function measured by the CSFQ-14, was found in the frequency of desire, in which a larger number of patients with epilepsy reported sexual dysfunction. In the studied cohort, sexual function in women is associated with both physical and mental health, and in men with physical health. These results should be considered when caring for patients with epilepsy and MS.

## Background

Living with a chronic disease affects quality of life, including physical, psychological, and social aspects. These aspects are closely connected, and if one part is affected it will most certainly influence the others [1–3]. Furthermore, chronic disease often influences the patient’s sexual life, mediated by bio-psycho-social factors related to the chronic disease. Multiple sclerosis (MS) and epilepsy are life-long neurological diseases in which sexuality may be affected, possibly resulting in sexual dysfunction. Chronic neurological diseases and medical treatment often have negative effects on sexuality and quality of life [1, 4]. Patients with MS or epilepsy are known to have increased sexual dysfunction, but the incidence varies according to the literature [5–7].

MS is a neurological disease affecting the central nervous system (CNS) via demyelination of the neurons and plaque formation. The prevalence is 50–300 per 100,000 people and is more common in people from northern Europe. The etiology of MS has not been clearly identified, but research suggests both a genetic and environmental context, such as vitamin D deficiency, diet, obesity in early life, and smoking [8]. MS usually manifests around 40 years of age, and predominantly among women (gender ratio 1:2) [9]. The course of the disease varies but can be quite progressive, despite treatment, and may result in different degrees of disability [9]. Treatments for MS can be divided into disease-modifying treatments, treatment of acute relapses, and management, including pharmacological and non-pharmacological treatment to alleviate symptoms, such as spasticity, bladder and bowel disorders, sexual disorders, paroxysmal phenomena, sclerosis-associated fatigue, and pain. Due to the location in the CNS, disabilities can be divided into specific symptoms (sensory disturbances, vision problems, and paralysis) or non-specific symptoms (cognitive symptoms such as memory and concentration difficulties, tiredness, and dizziness). Rees et al. showed that 50–75% of men with MS experience erectile problems, approximately 60% find it difficult to ejaculate, and approximately 40% have decreased sexual desire. The same review reported that 33% of women with MS have difficulties achieving orgasm, 27% have a loss of sexual desire, 36% experience decreased vaginal lubrication, and 12% experience increased spasticity during intercourse. For both genders, fatigue, depression, spasticity, and concern about incontinence are associated with an increase in sexual problems [10].

Epilepsy may debut at any point in life, though up to 75% of cases begin in childhood and older age [11]. The prevalence is equal in women and men, though slightly higher in boys from 0 to 5 years of age [12]. The etiology of epilepsy includes a wide range of underlying causes, such as genetic, CNS infection, head trauma, neurodegenerative, stroke, and CNS tumor [13]. Antiepileptic drugs (AEDs) control 70% of patients’ seizures, whereas the remaining 30% do not have complete seizure control with AEDs [11] [14]. If seizure control is not achieved with AEDs, surgery may be an option, and as many as 60–80% of patients gain seizure freedom [7]. The prevalence of sexual dysfunction in patients with refractory epilepsy has been estimated to be 31–64% in women with epilepsy and 64–91% in men [5]. A study from Norway reported the type of dysfunction in women and men to be 52 and 26% reduced sexual desire, 35 and 13% problems with orgasm, 27 and 1% pain during intercourse, and 4 and 6% late ejaculation, respectively, with vaginal dryness in 31% of women and premature ejaculation and erectile dysfunction in 16 and 34% of men, respectively [15]. Both epilepsy and AEDs affect hormones and the neuroendocrine system, resulting in disturbances in sexual responses, such as reduced desire, orgasmic and erectile dysfunction, and dissatisfaction from sexual intercourse [16]. Herzog et al. found significantly lower bioactive testosterone in men treated for epilepsy than in the control group, but the choice of pharmacological treatment also influences sexual function [17]. Medical treatment independently predisposes to sexual dysfunction in patients with MS and epilepsy. This is due in part to changes in the brain’s processing of sexual stimuli, resulting in decreased potency, absence of sexual arousal, problems with orgasm, or physiological changes with difficulties having intercourse [18]. Anti-epileptic drugs may affect sexual function both positively and negatively. Anti-convulsants which induce the cytochrome P450 enzyme system have high impact on sexual dysfunction, primarily by changes in sex hormone levels [14, 19]. Some newer anti-convulsants such as oxcarbazepine and lamotrigine may improve sexual function, which also needs to be taking into consideration when treating patients with epilepsy.

The primary aim of this study was to describe the prevalence of sexual dysfunction in patients with epilepsy and MS, and investigate whether there is an association between disease, sexual function, and physical and mental health. A secondary aim was to investigate whether there is a difference in sexual function between patients with MS and epilepsy. We hypothesize that patients with MS more frequently have sexual dysfunction compared to patients with epilepsy due to the often more profound consequences of the disease. Furthermore, we predict that sexual dysfunction is associated with impaired self-assessed physical health in men and impaired self-assessed mental health in females.

## Methods

**Fig. 1:**
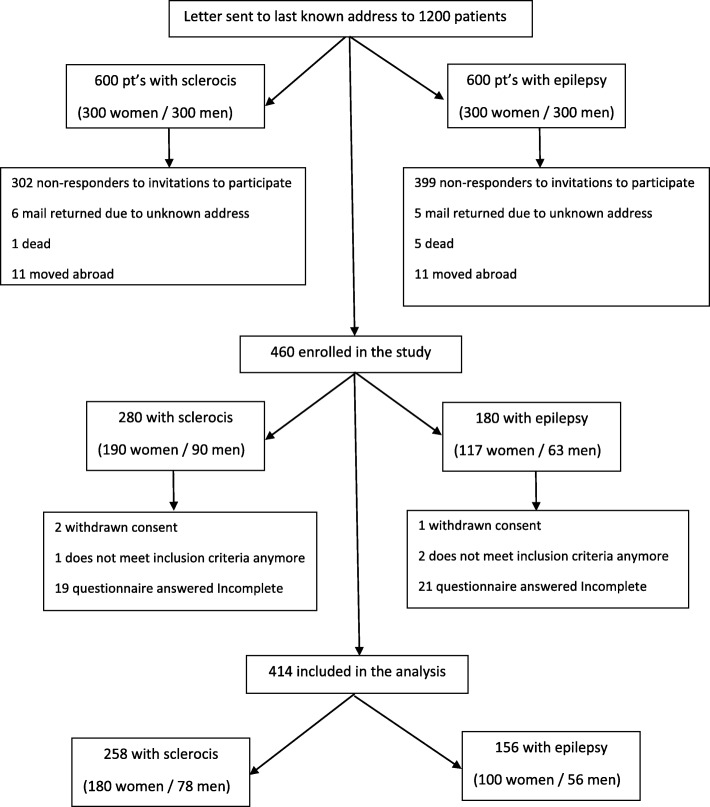
Flowchart

This study was a cross-sectional study of men and women followed at the Neurological clinic at Copenhagen University Hospital. Patients were randomly selected from date of birth (600 from each patient group) and invited to participate in the study as shown in Fig. 1. The invitation to participate in the study was sent together with questionnaires. If no response was received after 6 weeks, a reminder was sent. Patients who wanted to participate in the survey were asked to sign an informed consent form and return it with the questionnaires in an enclosed stamped envelope. The questionnaires were numbered consecutively so the responses were anonymous. Inclusion criteria were diagnosis with either multiple sclerosis or epilepsy and age ≥ 18 years. Exclusion criteria were an inability to complete the study, exacerbation of disease (hospitalized, substantial changes in disease) assessed by the study investigator, inability to understand and read Danish, or reduced cognitive function (e.g., diagnosis of dementia).

### Sample size

For the comparison between MS and epilepsy patients a significance level of 0.05 and power of 80% were used. We estimated the sexual dysfunction in patients with epilepsy to be 78% [5] and in patients with MS to be 63% [7]. Based on these estimates, a total of 288 participants were calculated to be needed, divided into two groups requiring 144 participants in each group.

### Primary measurement

The Changes in Sexual Function Questionnaire (CSFQ) is a validated 14-point instrument for measuring sexual function in females and males [20]. Scores are reported by a 5-point Likert scale that refers to either frequency (“never” to “every day”) or satisfaction (“none” to “great”). A cut-off score was calculated for the total CSFQ score (female ≤47, male ≤41). Furthermore, cut-off levels exist for the different subdomains: pleasure (female ≤4, male ≤4), desire/frequency (female ≤9, male ≤11), desire/interest (female ≤9, male ≤11), arousal/excitement (female ≤12, male ≤13), and orgasm/completion (female ≤11, male ≤13). A score equal to or below the cut-off point on any of the scales indicate possible sexual dysfunction. The CSFQ questionnaire has been validated in Danish.

### Secondary measurement

Two questionnaires were used to collect health and quality of life data. The Short Form 36 Health Survey (SF-36) is a validated multi-dimensional instrument measuring the health-related quality of life [21, 22]. The SF-36 assesses different aspects of health, level of function, and well-being of eight different dimensions: physical function, role-physical, bodily pain, general health, vitality, social function, role-emotional, and mental health. The eight dimensions can be grouped into a total SF score, a Physical Component Summary (PCS) including physical function, role-physical, bodily pain, and general health, and a Mental Component Summary (MCS) including vitality, social function, role-emotional, and mental health. The SF-36 was validated in Danish [23, 24]. In this study, the SF total score, PCS, and MCS were used to describe a subjective reported health score.

Life Satisfaction—11 (LiSat-11) is an 11-item self-reported questionnaire, with one question regarding general satisfaction with life and 10 questions regarding specific aspects. Satisfaction is scored on a scale from 1 to 6, with higher scores indicating greater satisfaction [25]. The 11 questions are reported in five domains: Life as a Whole, Closeness, Health, Spare Time, and Family Life.

### Other measurements included in the questionnaire

General demographic questions included gender, age at inclusion, age at diagnosis, disease duration in year, and partner status (married, living together, or seeing regularly). Socio-demographic questions included education ≥12 years (yes/no), employment (yes/no), and income ≥42,000 Euro. Information concerning patients’ medication was subcategorized into diagnosis-related medication, cardiovascular medication, psychopharmaceutic medication, and antidiabetic medication. Finally, data regarding bladder function was collected.

### Statistical analysis

Descriptive statistics (mean and standard deviation [SD]) were used to summarize the clinical and anthropometric data. Student’s t-tests and chi-square tests were used when comparing study data to reference subjects. Linear regression analysis using Enter was applied with the CSFQ total score as the dependent variable and SF-36 PCS, SF-36 MCS, Diagnosis, Partner, Age at project start, and Education ≥12 years as variables. All analyses were conducted using SPSS version 24. A significance level of *p* ≤ 0.05 was used for all statistical analyses.

### Ethics

Informed consent was obtained from each patient, and the study was approved by the Ethical Committee of Copenhagen (protocol H-15000601) and the Data Protection Agency (protocol 2014-41-3565).

## Results

Of the 1200 patients invited to participate in the study, 460 (38%) returned the questionnaires, 414 of which were completed adequately to be used in the analysis (Fig. 1). Of the 414 questionnaires, 32% (*n* = 134) were from men and 68% (*n* = 280) from women. Patients with MS constituted 62% (*n* = 258) of the final study population and patients with epilepsy 38% (*n* = 156). The median age of the whole population was 45 years. Patients with epilepsy were older than patients with MS (*p* = 0.001), whereas patients with MS were older when diagnosed (*p* = 0.000), patients with epilepsy had a significant higher disease duration (*p* = 0.000). A greater proportion of patients with MS had more than 12 years of education compared to patients with epilepsy (*p* = 0.001). No significant differences were found in gender, regular partner, or employment between patients with MS and epilepsy. There was a significant between the two groups regarding bladder dysfunction showing patients with MS to have more negative affected bladder function. Regarding medication, antidiabetics were more common among patients with epilepsy (p<0.05) (Table 1).

### CSFQ

Overall, 71% of the population had a cut-off indicating sexual dysfunction (Table 2). No differences were found between patients with MS and epilepsy (*p* = 0.184) except in the desire domain; patients with epilepsy more often had a desire problem than patients with MS (*p* = 0.029). When comparing gender, no differences were found in the CSFQ total score or the subdomains between patients with MS and epilepsy except for the desire domain; women with epilepsy had significant lower desire than women with MS (*p* = 0.020).

**Table 1:** Data including socio-demographic, Short Form 36 (SF-36), Life Satisfaction 11 (LiSat-11) for whole sample and comparison between patients with epilepsy and multiple sclerosis

	Total n (%)	Epilepsy n(%)	Multiple Sclerosis n (%)	*p*
Number	414 (100)	156 (38)	258 (62)	
Gender, male	134 (32)	56 (37)	78 (30)	.233
Age, median, range	45, 21–76	49,21–76	44, 21–73	.001
Age at diagnoses, median, range	30, 1–71	23, 1–71	30, 12–66	.000
Disease duration in age, median, range	13, 2–71	18, 2–71	12, 2–30	.000
Regular partner, Yes	307 (74)	110 (72)	197 (80)	.070
Education ≥12 years, Yes	301 (73)	99 (64)	202 (78)	.001
Employment at project start, Yes	269 (65)	95 (60)	174 (67)	159
Income ≥42.000 Euro, Yes	296 (72)	105 (67)	191 (74)	.160
Bladder dysfunction, Yes	198 (48)	27 (18)	171 (67)	.000
Medicated with				
*Diagnosis related medications*	371 (90)	140 (90)	231 (90)	.946
*Cardiovascular medication*	57 (14)	20 (13)	37 (15)	.622
*Psychopharmaca*	52 (13)	23 (15)	29 (12)	.323
*Antidiabetics*	14 (4)	9 (6)	5 (2)	.042
SF-36	*n* = 397 (mean (SD)	*n* = 144 (mean (SD)	*n* = 253 (mean (SD)	
*Total score*	76.6 (12.7)	79.9 (11.4)	74.9 (13.1)	.000^◊^
*Physical Component Summery*	41.6 (9.0)	44.9 (7.4)	39.8 (9.4)	.000^◊^
*Mental Component Summery*	49.8 (10.5)	47.6 (11.2)	51.0 (9.8)	.002^◊^
LiSat-11	*n* = 410 (mean (SD)	*n* = 154 (mean (SD)	*n* = 256 (mean (SD)	
*Life as a Whole*	4.8 (1.1)	4.7 (1.3)	4.8 (1.0)	.176^◊^
*ClosenessHealthSpare time*	4.2 (1.5)	4.2 (1.6)	4.3 (1.5)	.414^◊^
*Family life*	4.7 (1.0)	4.7 (1.1)	4.6 (1.0)	.679^◊^
	4.7 (1.0)	4.6 (1.3)	4.7 (1. 1)	.522^◊^
	4.5 (1.4)	4.4 (1.4)	4.6 (1.3)	.176^◊^

◊ = Student’s *t*-test, otherwise Chi-square test. SF-36 range up to 100, 100 is best. LiSat-11 range 1–6, 6 is best

Correlation analyses between use of medication for depression, CSFQ cut off respective for male and female, and diagnosis showed significant correlation between use of medication for depression and CSFQ cut off for male (.214, *p* = 0.050).

### SF-36

When measuring self-rated health-related quality of life using the SF-36, a significant difference was observed between the patients with MS and epilepsy (Table 2). In the PCS total score, patients with epilepsy scored significantly higher than patients with MS (*p* = 0.000), indicating that patients with MS had more adversely affected mobility. In contrast, patients with MS scored significantly higher on the MCS (*p* = 0.002), indicating that patients with epilepsy have adversely affected mental health.

**Table 2:** COSQ-14 cut off* scores for women and men and comparison when divided into Epilepsy and Multiple Sclerosis

		Both gender n (%)		Female n (%)			Male n (%)		
	Sexual Dysfunction	No Sexual Dysfunction	*p*	Sexual Dysfunction	No Sexual Dysfunction	*P*	Sexual Dysfunction	No Sexual Dysfunction	*P*
CSFQ Total, women cut off ≤41/ men cut off ≤47									
All	292 (71%)	122 (29)		189 (68)	91 (32)		103 (77)	31 (23)	
Epilepsy	116 (74%)	40 (26)		73 (73)	27 (27)		43 (77)	13 (23)	
Multiple sclerosis	176 (68%)	82 (32)	.184	116 (64)	64 (36)	.264	60 (77)	18 (23)	.985
Pleasure, women cut off ≤4/ men cut off ≤4									
All	313 (76%)	101 (24)		209 (75)	71 (25)		104 (78)	30 (23)	
Epilepsy	125 (80%)	31 (20)		78 (78)	22 (22)		47 (84)	9 (16)	
Multiple sclerosis	188 (73%)	70 (27)	.096	131 (73)	49 (27)	.336	57 (87)	21 (13)	.137
Desire/Frequency, women cut off ≤6/ men cut off ≤8									
All	333 (80%)	81 (20)		216 (77)	64 (23)		117 (87)	17 (13)	
Epilepsy	134 (86%)	22 (14)		85 (85)	15 (15)		49 (86)	7 (14)	.956
Multiple sclerosis	199 (77%)	59 (23)	.029	131 (73)	49 (27)	.020	68 (87)	10 (13)	
Desire/Interest, women cut off ≤9/ men cut off ≤11									
All	301 (73%)	113 (27)		204 (73)	76 (27)		97 (72)	37 (28)	
Epilepsy	118 (76%)	38 (24)		76 (76)	24 (24)		42 (75)	14 (25)	
Multiple sclerosis	183 (71%)	75 (29)	.297	128 (71)	52 (29)	.378	55 (71)	23 (29)	.567
Arousal/Excitement, women cut off ≤12/ men cut off ≤ 13									
All	339 (82%)	75 (18)		237 (85)	43 (15)		102 (76)	32 (24)	
Epilepsy	126 (81%)	30 (19)		86 (76)	14 (14)		40 (71)	16 (29)	
Multiple sclerosis	213 (83%)	45 (17)	.647	151 (84)	52 (16)	.639	62 (79)	16 (21)	.281
Orgasm/Completion, women cut off ≤11/ men cut off ≤13									
All	301 (73%)	113 (27)		190 (68)	90 (32)		111 (83)	23 (17)	
Epilepsy	128 (82%)	28 (18)		72 (72)	28 (28)		47 (84)	9 (16)	
Multiple sclerosis	196 (76%)	62 (24	.146	118 (66)	62 (34)	.269	64 (82)	14 (18)	.776

*A score at or below cut off point on any scales indicate sexual dysfunction, *p* = Chi-square test

### LiSat-11

Patients with MS scored just a little higher in four out of five domains on LiSat-11, but no significant differences were found between the MS and epilepsy patients (Table 2).

### Regression analysis

Among men, the CSFQ total score was significantly associated with SF-36 PCS (*t* = 3.398, *p* = 0.001), having a partner (*t* = 3.381, *p* = 0.001), and age (*t* = − 4.607, *p* = 0.000), but no significant association was found with SF-36 MCS, diagnosis, or education. In women, the CSFQ total score was significantly associated with SF-36 PCS (*t* = 3.556, *p* = 0.000), SF-36 MCS (*t* = 3.039, *p* = 0.003), diagnosis (*t* = 2.103, *p* = 0.036), having a partner (*t* = 3.482, *p* = 0.001), and age (*t* = − 4.223, *p* = 0.000). Disease duration and education had no significant association on the CSFQ total score (Tables 3 and 4).

**Table 3:** Linear Regression analysis included all male

*n* = 134	t	Sig.
SF-36, Physical Component Summery	2.981	.004
SF-36 Mental Component Summery	.171	.865
Diagnosis	.338	.736
Partner	.870	.005
Age	−3.266	.002
Education ≥12 years	−.522	.603
Disease duration in year	1.608	.111

Dependent Variable: CSFQ Total score in male

**Table 4:** Linear Regression analysis included all female

*n* = 280	t	Sig.
SF-36, Physical Component Summery	2.074	.039
SF-36 Mental Component Summery	.923	.357
Diagnosis	2.040	.043
Partner	2.657	.009
Age	−3.513	.001
Education ≥12 years	1.076	.283
Disease duration in year	.884	.378

Dependent Variable: CSFQ Total score in female

## Discussion

The primary aim of this study was to investigate the prevalence of sexual dysfunction in patients with epilepsy or multiple sclerosis. Overall, 68% of women and 77% of men reported possible sexual dysfunction measured by the total CSFQ. In each of the five CSFQ domains, both women and men had highly negatively affected sexual function. The study found women had the most difficulty in the domains arousal/excitement, and men had most difficulties in the domains desire/frequency. Previous studies investigating background population have shown that women have affected desire, arousal difficulty, and sexual pain and men have problems with premature ejaculation, low sexual desire, and erectile dysfunction [2, 26, 27]. In a comparative survey including 171 patients with epilepsy and 593 subjects from the general population, Henning et al. found significant differences between the general population and patients with epilepsy regarding sexual problems and sexual dissatisfaction [15]. Similarly, Atif et al. found that women with epilepsy report reduced sexual arousability and men reported erectile dysfunction as the worst problems [28]. In a survey including 4415 participants, Christensen et al. investigated associations between physical and mental health problems in sexually active Danes and found that physical health problems are significantly associated with sexual dysfunction in men and mental health problems are significantly associated with sexual dysfunction in women [27] . One of the mechanisms by which sexual function is impaired by neurological disease may be a negative impact on activation of the limbic and paralimbic regions responsible for sexual, emotional, and motor responses as described by Rees et al. [10].

When comparing patients with MS and epilepsy in the present study, both groups reported a high level of sexual dysfunction. Only in the domain desire/frequency was a significant difference was found between the groups, as women with MS had a more negative score. This difference is difficult to explain, but one can speculate that women with epilepsy have an earlier start of disease and might have gotten more “used” to a lower libido, such to a lesser degree registering it as a problem. Patients with MS may have higher negatively affected immobility, as shown in a longitudinal study by Young et al. that included 538 patients, finding a close link between MS and the level of expanded disability [29]. Desire may decrease when the patient experiences difficulties concerning the psychical part of sexual intercourse. In a clinic-based study including 100 patients, Celik et al. found no significant gender differences in patients with MS regarding fear of being rejected, worries about sexually satisfying their partner, or lack of confidence about their sexuality [30].

Other factors may complicate the sexual lives of patients with epilepsy. In a qualitative study including 26 patients, Egerod et al. found that patients with epilepsy feared an epileptic seizure if they let go during sexual activity and, therefore, do not achieve satisfaction and/or orgasm by intimate contact [31] . This was supported by Rees et al. in describing sexual auras and erotic feeling as part of seizures [10]. In the present study, it is not possible to say anything about the underlying mechanisms, but 82% of the patients experienced orgasmic problems that could be due to these reasons. For both patient groups, mental factors such as fatigue, low self-esteem, and fear of incontinence during sexual intercourse have been reported to affect sexual desire [18].

No significant difference was found in LiSat-11 scores between the two patient groups, though both groups of patients had negatively affected quality of life. Our results are in line with other studies. In a focus group investigation of sexual health in people with MS and epilepsy, Egerod et al. found that sexuality was affected in both groups and sexual activity was closely related to quality of life [31]. In a dataset of message board posts from the Epilepsy Foundation of America website, Miller et al. found 2015 posts concerning challenges that patients with epilepsy experience in relation to sexuality, such as sexual desire, shame/embarrassment, epilepsy-driven relationship deterioration, and sex-related syndromes [32]. A registry study including 6183 patients investigated the impact of sexual dysfunction on health-related quality of life in patients with MS and found that both mental health and physical disability have a negative impact on quality of life [33].

Patients with MS scored significantly lower on the SF-36 total score and PCS, whereas patients with epilepsy scored lower on the PCS, sustaining the results from LiSat-11. Even though we found that patients with MS have no significant problems with the mental component, other studies have shown that quality of life in patients with MS is influenced by mental health. In a review of sexual problems among men with MS, Calabró et al. concluded that they were influenced not only by their physical being, but by mental health, and that their quality of life has an impact on sexuality as well [7]. Egerod et al. also found that patients with MS and epilepsy experience a negative mental impact due to the disease [31]. In a cross-sectional study including 506 patients, Mameniskiene et al. found that patients with epilepsy have a negatively affected family life and experience social stigma [34] . Furthermore, patients with epilepsy need help empowering the challenges of living with a chronic disease such as epilepsy.

In the present study, we found that several factors influenced sexual function in patients with MS and epilepsy. Having a regular partner was found to have a positive impact on sexuality in both men and women. This finding is in line with a study in a nationally representative Danish population; in both men and women, being married or cohabitant correlated with having sexual desire [35]. Studies have shown that not being married correlates to men having erectile dysfunction, and in women low desire correlates with having a partner [36]. This can be explained by the fact that having a partner results in one more being confronted with the impaired sexual function and makes the patient more distressed by the problem compared to if one is alone. In addition, a problem such as erectile dysfunction can prevent one from finding a partner. The positive effect of having a partner is described by Basson et al. for patients with neurological diseases, as having someone to share concerns and thoughts with may improve the relationship [37]. Jennum et al. found that approximately 50% of patients with epilepsy were living alone [12]. This is in contrast to our findings that 77% of the patients with epilepsy and 82% of the patients with MS had a regular partner. One explanation for the higher frequency in our study may be that patients who have a partner are more prone to participate in the study. Participating in the study may be encouraged by the partner; thus, the patients’ and partners’ sexuality is the driving force for clarifying solutions to sexual dysfunction, which may support the assumption that having a regular partner helps patients with a chronic disorder be more open to conversation about their sexual difficulties.

In both genders, age had a significant negative impact on sexuality. The fact that sexual activity changes with increasing age is well known, and the experience gained through life has an influence on expectations [2, 26, 36]. In a review, McCabe et al. found that age negatively influences sexuality, but also that comorbidity and healthy living are essential for how elderly deal with sexuality [38] Eplov et al. found that sexual desire decreases with increasing age, and around 50% reported a decline in the level of sexual desire that stopped around 50 years.

Most of the participants in our study used medicine related to their neurological condition, and this may have an impact on sexual function. Depending on the specific drug, the treatment of epilepsy may result in sexual hormone changes, psychological factors, and neurotransmitter disorders that present as reduced desire, orgasmic dysfunction, erectile dysfunction, and dissatisfaction from sexual intercourse [5, 10, 14, 16]. Herzog et al. found a significant negative effect on bioactive testosterone in men treated for epilepsy, resulting in sexual dysfunction [17]. In addition, co-morbidities treated with cardiovascular medication, psychopharmaceuticals, and antidiabetics are known to affect sexual function. In a review of erectile dysfunction in cardiovascular patients, Vlachopoulos et al. found a negative effect of high statin doses, and a potential association with reduced serum testosterone levels [39]. Montejo et al. found that both antidepressants with serotonergic activity and antipsychotics increase prolactin levels and block dopamine receptors and are related to sexual dysfunction [40]. A review by Hackett stressed that low levels of testosterone frequently seen in men receiving diabetic and testosterone therapy seem to benefit sexual function and quality of life [41] . In the present study, the levels of testosterone were not investigated; therefore, it is difficult to determine if low testosterone affects sexuality.

Overall, the present study shows that patients with MS and epilepsy report a high prevalence of sexual dysfunction. The results support the findings from a previous focus group investigation reporting that patients seldom highlight sexual problems when talking to health professionals, which may result in sexual distress and impact both psychological wellbeing and the relationship with a partner [31]. Some sexual problems may be related to the current disease, whereas other sexual problems may be triggered by more psychological and social aspects. It is important to discuss a disease’s potential impact on sexuality with the patients. The present study emphasizes the importance of the health care provider discussing sexual function with patients with MS and epilepsy [4, 31, 37].

### Strengths and limitations

A strength of the present study is the large number of participants and the possibility of comparing two different groups of patients with neurological diseases. The limitations of this study include the lack of a control group with no disease, potential selection bias, creating the risk of a type II error, as it is possible patients having a partner and/or social life more often chose to participate. Patients living alone and/or without a social network may have failed to participate in the study and, thus, are underrepresented. Another limitation of the study is the lack of precise knowledge regarding types of medication. Medications used in the treatment of either MS or epilepsy have different impacts on sexuality and specific knowledge about the medication may have clarified the results even more. Unfortunately, data on state of MD and type of epilepsy were not collected although, this knowledge would have strengthened the outcome of the study and future studies should include these information’s. Furthermore, we did not use a specific disability score to characterize the patients. The rather small responder rate should be taken into consideration if using the results in the clinic, though all patients may benefit from having a dialog about sexuality and sexual function regarding the disease.

## Conclusions

The cohort of patients with MS and epilepsy in this study have negatively affected sexuality. The only significant difference between the two patient groups in sexuality measured by the CSFQ-14 was the frequency of desire, with more patients with epilepsy reporting sexual dysfunction, but the neurological diagnosis did not seem to predict the degree of sexual dysfunction. Patients with MS scored worse in physical health, and patients with epilepsy scored worse in metal health. Sexuality in women was associated with both physical and mental health. Sexuality in men was associated with physical health. These results should be considered when talking with patients about potential sexual problems and solutions.

## Data Availability

The data generated during the current study are not publicly available. The are available from the corresponding author on reasonable request.

## Abbreviations

AED: AntiEpileptic Drugs
CNS: Central Nervous System
CSFQ: Changes in Sexual Function Questionnaire
LiSat-11: Life Satisfaction—11
MCS: Mental Component Summary
MS: Multiple Sclerosis
PCS: Physical Component Summary
SF-36: Short Form 36 Health Survey
